# School knowledge of infectious diseases in schools: conducting surveillance and on-demand, symptomatic respiratory viral testing in a large pre-kindergarten–12th grade school district

**DOI:** 10.3389/fpubh.2024.1408281

**Published:** 2024-07-23

**Authors:** Jennifer E. Schuster, Tamoor T. Chohdry, Chris T. Young, Brian R. Lee, Dithi Banerjee, Anjana Sasidharan, Olivia M. Almendares, Hannah L. Kirking, Janelle Porter, Anila Deliu, Shannon Tilsworth, Rangaraj Selvarangan, Jennifer L. Goldman

**Affiliations:** ^1^Children’s Mercy Kansas City, Kansas City, MO, United States; ^2^Coronavirus and Other Respiratory Viruses Division, National Center for Immunization and Respiratory Diseases, Centers for Disease Control and Prevention, Atlanta, GA, United States; ^3^North Kansas City Schools, Kansas City, MO, United States

**Keywords:** acute respiratory illness, respiratory virus, school, students, teachers, surveillance

## Abstract

**Background:**

Limited data about acute respiratory illness (ARI) and respiratory virus circulation are available in congregate community settings, specifically schools. To better characterize the epidemiology of ARI and respiratory viruses in schools, we developed School Knowledge of Infectious Diseases in Schools (School KIDS).

**Methods:**

School KIDS is a prospective, respiratory viral testing program in a large metropolitan school district (pre-kindergarten–12th grade) in Kansas City, Missouri. During the 2022–2023 school year, all students and staff were eligible to participate in surveillance respiratory viral testing at school by submitting observed self-administered nasal swabs monthly. Participants could also submit a nasal swab for on-demand symptomatic testing when experiencing ≥1 ARI symptom, including cough, fever, nasal congestion, runny nose, shortness of breath, sore throat, and/or wheezing. Swabs were tested in a research laboratory using multipathogen respiratory polymerase chain reaction assays. Participants were evaluated for ongoing viral shedding by collecting two weekly nasal swabs (i.e., convalescent), following initial on-demand symptomatic testing. Participants were asked to complete an electronic survey to capture the presence and type of ARI symptom(s) before the collection of respiratory swabs.

**Results:**

From 31 October 2022 to 29 June 2023, School KIDS enrolled 978 participants, including 700 students, representing 3.4% of the district student population, and 278 staff members. Participants submitted a median of six surveillance, one symptomatic, and two convalescent specimens during the study period. A total of 6,315 respiratory specimens, including 4,700 surveillance, 721 on-demand symptomatic, and 894 convalescent specimens, were tested. Overall, a virus was detected in 1,168 (24.9%) surveillance and 363 (50.3%) symptomatic specimens. Of the 5,538 symptom surveys sent to participants before scheduled surveillance testing, 4,069 (73.5%) were completed; ARI symptoms were reported on 1,348 (33.1%) surveys.

**Conclusion:**

Respiratory surveillance testing in schools is feasible and provides novel information about respiratory virus detections in students and staff attending school. Schools are an important community setting, and better knowledge of respiratory virus circulation in schools may be useful to identify respiratory virus transmission in the community and assess the impact of effective infection prevention measures.

## Introduction

Acute respiratory illnesses (ARIs) are a major burden in children (1). Active clinical surveillance mechanisms have been used to describe medically attended rates of ARI and associated respiratory viruses (2). However, most ARIs do not result in children seeking medical attention (3, 4), thus non-medical (e.g., community) surveillance systems are needed to better understand the epidemiology of ARI. Schools are unique community settings, and school-based surveillance for respiratory viruses has broad implications, including the ability to assess the burden of non-medically attended ARI and identify the pathogens resulting in non-medically attended ARI. Additional public health impacts could include understanding who is a high-risk individual for infection in the school setting, the impact of vaccination on non-medically attended ARI caused by respiratory viruses, how respiratory viral transmission may occur in schools, and the impact of school-based mitigation measures to reduce transmission (e.g., upgraded ventilation). School-based surveillance could serve as an early indicator for broader community transmission prior to medically attended surveillance platforms (5). Despite the potential benefits, strategies for implementing successful school-based ARI and respiratory viral surveillance systems have not been well established.

Previously described school-based surveillance systems have mostly utilized absenteeism or syndromic surveillance as a correlate of ARI in the community (5–9). However, these studies have been primarily conducted during influenza season, focused on influenza-like illness, and correlated with seasonal influenza rates in the community or hospital, thus lacking a multipathogen focus. In a daycare ARI surveillance study, rhinovirus, adenovirus, and seasonal coronaviruses were the most common pathogens detected during ARI episodes (10). These data suggest that the epidemiology of viral ARI in congregate educational settings is diverse; however, limited data are available in kindergarten (K)–12th grade students. This lack of data related to the baseline circulation of respiratory viruses in the school setting resulted in a knowledge gap about the role of schools in respiratory virus infections and outbreaks during the COVID-19 pandemic. Widening the lens through which we understand viral ARI using broader, multipathogen microbiologic testing provides an opportunity to better understand the epidemiology of ARI in school settings.

Performing school-based ARI and viral surveillance has unique challenges compared with hospital and clinic-focused surveillance systems, and recent attempts to implement school-based SARS-CoV-2 surveillance programs during the COVID-19 pandemic provide valuable information on potential barriers. Although resources (e.g., testing and personnel) were available to these programs, participation was poor, highlighting the difficulty of instituting testing programs in schools (11). Barriers to enrollment included the need for parent/legal guardian consent in a setting with limited parental access, the use of an invasive test procedure (i.e., nasopharyngeal respiratory swabs), potential consequences of viral detection (i.e., isolation and inability to attend in-person learning), and interruption of the school day when testing was performed (12). Different strategies are needed to engage and work with school communities to successfully perform ARI surveillance and understand viral infections in schools.

Successful implementation of school-based respiratory virus surveillance could provide useful information for community circulation of respiratory viruses and elucidate the role of schools in future epidemics and pandemics. Therefore, to better understand the epidemiology of ARI and respiratory viruses in schools, we designed and implemented School Knowledge of Infectious Diseases in Schools (School KIDS), a longitudinal study to test pre-K–12th grade students and school staff for respiratory viruses in the school setting and assess associated symptoms. The objective of this study is to describe rates of symptomatic and asymptomatic viral detection in the school setting with the aim of increasing the understanding of ARI and respiratory virus prevention and viral transmission in the school setting. Here, we describe the implementation and high-level results from the program including participation rates, characteristics of enrolled participants, assessment of symptomatology, the total number of respiratory samples collected, and viral detections.

## Materials and methods

### Study location

The North Kansas City School District (NKCSD) spans across 82 square miles and is located primarily in Clay County, Missouri, along with smaller portions of Jackson and Platte counties. The NKCSD is composed of 33 traditional public school buildings, namely, 1 early childhood center, 22 elementary (pre-K and kindergarten–5th grade), 6 middle schools (two schools with sixth grade only and four schools with both 7th and 8th grades), and 4 high schools (9th–12th grades).

### Study design

The protocol for this evaluation was reviewed by both Children’s Mercy Kansas City (CMKC) and the Centers for Disease Control and Prevention (CDC) and was conducted consistent with applicable federal law and CDC policy as defined 45 CFR 46.102(I)(2) (13). A data use agreement between NKCSD and CMKC allowed for bi-directional data sharing in a manner compliant with both the Health Insurance Portability and Accountability Act (14) and the Family Educational Rights and Privacy Act (15). Students were eligible to participate in School KIDS if they attended school in the NKCSD as were all staff members. Students/staff chose to participate in either on-demand symptomatic respiratory testing only (e.g., parent/participant requested testing when the participant was sick with ARI symptoms) or both on-demand symptomatic and surveillance respiratory testing (e.g., monthly testing occurring while in school) (Supplementary Figure S1). Participants who submitted an on-demand symptomatic specimen were also scheduled for weekly convalescent testing in the subsequent 2 weeks to estimate viral shedding over time. Once enrolled, participants in the surveillance respiratory testing were followed longitudinally over the course of the school year. Participants in on-demand symptomatic testing could submit additional specimens throughout the school year but were not actively followed. Participants who enrolled in surveillance testing were tested during the next scheduled school visit. Participants who enrolled in on-demand symptomatic were able to submit a test anytime after enrollment if symptoms were present.

For each specimen collected, participants received $15 on a reloadable gift card as compensation for their time. Participants, parents/ legal guardians, and school district staff were able to access the study team with any questions by email or phone during regular school hours.

### Enrollment

Enrollment began on 31 October 2022, in eight pilot schools with respiratory viral testing starting on 9 November 2022. Enrollment expanded to the remaining 25 schools on 30 November 2022. Rolling enrollment continued throughout the investigation with samples collected up to 30 June 2023, which included summer school. Communication materials used for opening enrollment and disseminating information for the School KIDS program consisted of a webpage and program flyers. The district and individual schools included information in their regular communications, including weekly newsletters and district-wide communications. Participation was optional with participants ≥18 years old and parents/legal guardians of participants <18 years old required to complete a Research Electronic Data Capture (REDCap) (16, 17) consent form, accessible by link and QR code. At the time of enrollment, a welcome letter with a study identification number (Supplementary Figure S2A), a home test kit (i.e., nasal swab, universal transport media, and biohazard bag), instructions on how to obtain and return the specimen (Supplementary Figure S2B), and reloadable gift card were mailed to participants.

### Data collection

At the time of enrollment, parents/participants completed an enrollment form that included the participant’s name, date of birth, address, school, grade (for students only), race, ethnicity, gender identity, language(s) spoken at home, number of people living in the primary household, and free and reduced lunch eligibility (for students only). The specific role of staff at school was not collected. They also selected their preferred method of communication (i.e., text message or email) and provided permission to obtain COVID-19 and influenza vaccination records from administrative providers. Participant data were collected and managed using REDCap data capture tools hosted at CMKC (16, 17).

### Symptom assessment

For surveillance and convalescent testing, a REDCap survey was sent to each participant 2 days before scheduled testing. This survey captured information on the presence/absence of ARI symptoms (including cough, fever, nasal congestion, runny nose, shortness of breath, sore throat, or wheezing) in the previous 7 days and whether symptoms were resolved or ongoing at the time the survey was completed. If the survey was not completed, it was resent two additional times, 12 h apart. The convalescent test survey also inquired whether the participant had sought medical attention for their illness. Surveillance and convalescent surveys also served the purpose of notifying the participant or parent/legal guardian that the participant would be tested that week in school.

For on-demand symptomatic testing, an assessment of symptoms occurred at the time testing was performed. Participants or parents/legal guardians completed an electronic specimen collection form (Microsoft Forms, hosted by CMKC), which included information about the date of onset and type of ARI symptoms, the date of specimen collection, and the method of specimen return to CMKC.

### Specimen collection

Specimens were collected using flocked swabs (Copan FLOQSwabs^®^ 502CS01, Murrieta, CA) inserted into the bilateral anterior nares and then placed in 3 mL of universal transport media (Copan UTM^®^: Viral Transport 3C047N, Murrieta, CA). Typically, swabs were self-collected but could be collected or assisted by the study team (for surveillance and convalescent), school nurse (for surveillance, convalescent, and on-demand symptomatic), or parent (for on-demand symptomatic). Participants and school nurses were instructed to keep the specimen in a refrigerator until the specimen could be transported, either by courier or by the study team, to CMKC, within 24 h after the specimen was obtained.

#### Surveillance specimen collection

Each school was scheduled for a regular surveillance testing day and time based on school nurse preference (e.g., daily school schedule and nurse availability); this was modified, as needed, if students were not at school or a large portion of students were unavailable (e.g., holidays, inclement weather days, field trips, and state testing). Surveillance collection was completed at the end of the school year and did not include summer school. Based on the number of participants enrolled at each school, school-level sample collection visits occurred monthly, biweekly, or weekly, with participants assigned to test at least monthly. School testing schedules, including date, time, and participants, were generated 1 week before testing to facilitate communication and awareness with school staff and participants/parents; this information was provided to a designated school contact person, either the school nurse or an office staff member. Two study staff members supervised specimen collection; participants were either called to the nurse’s office, or study staff went to the participant’s location (e.g., classroom). Participants in class were asked to step out briefly. If students or staff were offsite or otherwise unavailable during the day of scheduled specimen collection, the sample could be obtained the day prior by the school nurse. If an on-demand symptomatic swab was submitted within a week before scheduled surveillance swab collection, any scheduled surveillance specimen was not collected. All surveillance swabs were obtained, while the participant was at school.

#### On-demand symptomatic specimen collection

Participants were also able to request on-demand testing during any respiratory illness. To be eligible for on-demand symptomatic testing, participants had to have more than one of the following symptoms: cough, fever, nasal congestion, runny nose, shortness of breath, sore throat, or wheezing. Specimen collection could occur at school (via nurse or study staff) or at home using the pre-provided test kit sent at the time of enrollment or at school with test kits provided to the school nurse. On-demand symptomatic specimen collection could occur throughout the study period, including summer school. If an on-demand symptomatic specimen was obtained outside of the school setting, participants could return the specimen to the study laboratory by calling a courier (instructions provided to the participant at the time of enrollment with no cost to the participant), bringing the specimen to any NKC school during regular school hours, or bringing the specimen to a CMKC Urgent Care located within the NKCSD boundaries (Supplementary Figure S2B). Specimens obtained at home and brought to an NKC school or obtained at school were couriered to the CMKC using the same commercial courier. Specimens brought to the CMKC Urgent Care were brought to CMKC using the hospital courier system, which ran twice daily. A new test kit was mailed to participants after their home test kit was used.

#### Convalescent specimen collection

To determine the duration of viral shedding, participants who submitted an on-demand symptomatic specimen were scheduled for weekly testing (i.e., convalescent) for the subsequent 2 weeks. Convalescent specimen collection occurred at school during scheduled surveillance testing times using the same procedures described above for surveillance specimen collection. For students receiving on-demand symptomatic testing before winter break, test kits were mailed to the participant’s residence with instructions to perform one convalescent test during the school break.

### Laboratory testing

All specimens were tested for adenovirus (AdV), human metapneumovirus (HMPV), influenza (Flu) A and B, parainfluenza viruses 1–4 (PIV 1–4), respiratory syncytial virus (RSV), rhinovirus/enterovirus (RV/EV), SARS-CoV-2, and seasonal coronaviruses 229E, HKU1, NL63, and OC43 (sCoV).

For surveillance and convalescent specimens, testing was performed in batch mode using the Panther Fusion System^®^ (Hologic, Inc., Marlborough, MA) and four Panther Fusion® mini-panel respiratory assays: *Flu A/B/RSV*, *Paraflu*, *SARS-CoV-2*, and *AdV/hMPV/RV*. A fifth mini-panel on an open channel was created using sCoV analyte-specific reagents from Panther Fusion. Additional assays included EV-D68 and specimen quality control. Cycle threshold values for all targets were obtained.

For on-demand symptomatic testing, QIAstat-Dx respiratory SARS-CoV-2 panel assay (QIAGEN, Germantown, MD) was used to provide the results to participants within 1 business day. All sample preparation and assay testing steps were performed within the QIAstat-Dx assay cartridge. Virologic test results (AdV, Flu A, Flu B, HMPV, PIV 1–4, RSV, RV/EV, SARS-CoV-2, and sCoV) and cycle threshold values were obtained for all pathogens.

### Result communication

The results of on-demand symptomatic testing were provided to the participant or parent/ legal guardian using their preferred method of communication. Notifications were sent informing that no virus was detected, influenza was detected, SARS-CoV-2 was detected, or a virus was detected that was not influenza or SARS-CoV-2. The results of surveillance and convalescent tests were not provided as specimens were not tested in real time. Aggregate results were provided to NKCSD and CDC via an electronic dashboard and regular reports.

### COVID-19 and influenza vaccination verification

At the time of enrollment, participants and parents/legal guardians consented to influenza and COVID-19 vaccine records being collected from state registries, COVID-19 vaccination cards, and school and CMKC records. Participants and parents/legal guardians could also agree for the study team to contact the participant’s provider to obtain vaccine records and were asked to provide vaccine provider contact information. At the end of the school year, vaccine verification was performed in the following order: (1) Kansas and Missouri immunization registries, (2) CMKC’s electronic medical record, and (3) participant provided locations. Sources were queried for administration of bivalent COVID-19 vaccine and 2022–2023 seasonal influenza vaccine. Once both vaccine types were identified, additional searches were not performed (e.g., if bivalent COVID-19 and 2022–23 influenza were found in the state registry, the vaccine provider was not contacted).

### Analysis

Descriptive statistics were used to describe participants, respiratory testing numbers, respiratory testing results, and survey responses. Data from the Missouri Department of Elementary and Secondary Education (18) and American Community Survey 1-year Estimates (19) were used to compare the gender, race, ethnicity, and social vulnerability index of participants in School KIDS (20) to the general NKCSD student body, Missouri population, and United States population. The percent positivity by specimen type (e.g., surveillance, on-demand symptomatic, and convalescent) and age group was calculated as was the proportion of virus detection by virus and age group. Vaccination status was determined for participants who received any COVID-19 vaccine, the bivalent COVID-19 vaccine, and the 2022–2023 influenza vaccine. Viral detection by epidemiologic week from School KIDS specimens was compared with the Missouri data from the National Respiratory and Enteric Virus Surveillance System (NREVSS) (21).

## Results

### Participants, enrollment, and retention

**Figure 1:**
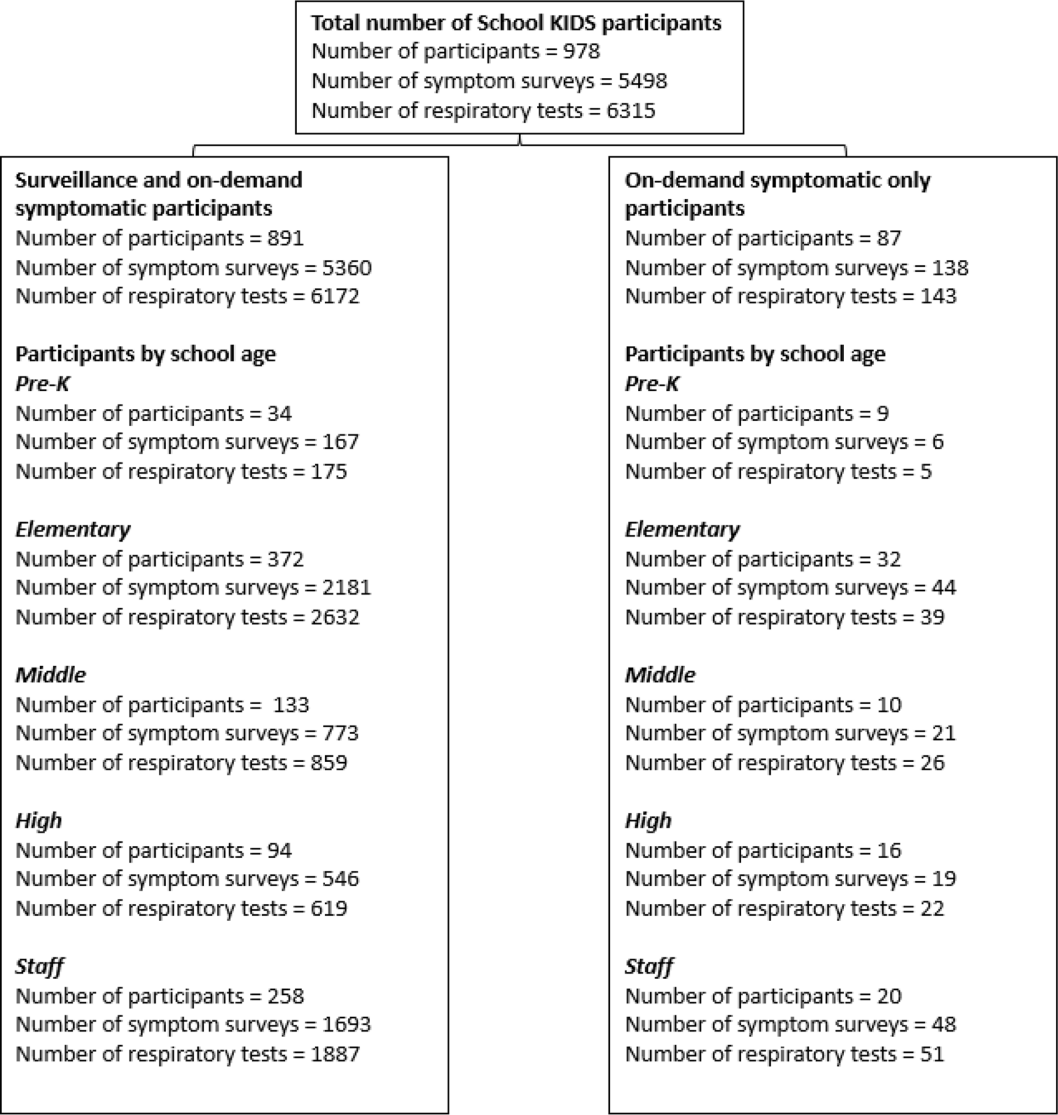
Numbers of School KIDS participants, surveys, and respiratory viral tests by type of enrollment (Kansas City, Missouri) 2022–2023.

From 31 October 2022 to 30 June 2023, 978 participants enrolled in School KIDS, a school-based viral detection surveillance study (Figure 1). Participants included 700 students, representing 3.4% of the total NKCSD students enrolled, and 278 staff. Overall, the highest participation was from elementary students and staff. The primary work location of staff enrollees included early childhood center (*n* = 9), elementary school (*n* = 188), middle school (*n* = 43), high school (*n* = 31), and other (i.e., district-level staff without a specific building location) (*n* = 7). Of all participants, 891 (91.1%) were enrolled in both surveillance and on-demand symptomatic arm [633 (90.4%) students and 258 (92.8%) staff], and 87 (9.9%) were enrolled in the on-demand symptomatic only arm [67 (9.6%) students and 20 (7.2%) staff]. The median duration of enrollment was 5.6 months [IQR 5.2, 5.7].

**Figure 2:**
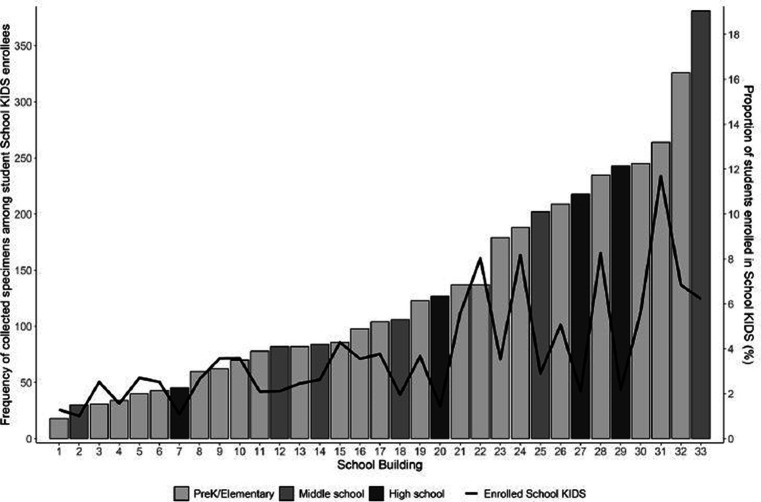
Number of collected specimens among student School KIDS enrollees and proportion of students enrolled.

Student enrollees (*n* = 700) included 43 (6.1%) pre-kindergarten, 404 (57.7%) elementary, 143 (20.4%) middle, and 110 (15.7%) high school students. Among student participants, 347 (49.6%) identified as female participants, 437 (62.4%) identified as white non-Hispanic (NH), 92 (13.1%) identified as Hispanic/ Latino, 52(7.4%) identified as Black NH, and 52 (7.4%) identified as Multi-racial NH (Table 1). Overall, School KIDS participant demographics were similar to Missouri and U.S. populations. The percentage of student enrollment by school ranged from 1.0 to 11.7% (Figure 2). Eight schools, namely, seven elementary and one middle school, had an enrollment that was ≥5% of the student population. Five schools, namely, two elementary, one middle, and two high schools, had an enrollment that was <2% enrollment of the student population. Differences were noted between demographics of School KIDS students and staff, with staff being majority female and white, NH.

**Table 1:** Demographics of School KIDS participants (Kansas City, Missouri) compared with local, state, and national data, 2022–2023.

	School KIDS participants (*n* = 978)	School KIDS staff (*n* = 278)	School KIDS students (n = 700)	NKCSD students (*n* = 20,419)^1^	Missouri population^2^ (*n* = 6,177,957)	United States population^2^ (*n* = 333,287,562)
Gender						
Female	593 (60.6%)	246 (88.5%)	347 (49.6%)	Not available^3^	3,131,606 (50.7%)	168,059,348 (50.4%)
Male	343 (35.1%)	22 (7.9%)	321 (45.9%)	Not available^3^	3,046,351 (49.3%)	165,228,214 (49.6%)
Other/Unknown	42 (4.3%)	10 (3.6%)	32 (4.6%)	Not available^3^	Not available^3^	Not available^3^
Race and ethnicity^4^						
White, non-Hispanic (NH)	663 (67.8%)	226 (81.3%)	437 (62.4%)	10,927 (53.5%)	4,733,411 (76.6%)	192,153,076 (57.7%)
Hispanic or Latino	112 (11.4%)	20 (7.2%)	92 (13.1%)	3,068 (15.0%)	291,763 (4.7%)	63,553,639 (19.1%)
Refused/Unknown	63 (6.4%)	13 (4.7%)	50 (7.1%)	Not available^3^	Not available^3^	Not available^3^
Black, NH	60 (6.1%)	8 (2.9%)	52 (7.4%)	3,154 (15.4%)	656,739 (10.6%)	39,582,961 (11.9%)
Multi-racial, NH	59 (6.0%)	7 (2.5%)	52 (7.4%)	2,256 (11.0%)	224,869 (3.6%)	11,063,758 (3.3%)
Asian, NH	15 (1.5%)	2 (0.7%)	13 (1.9%)	689 (3.4%)	132,436 (2.1%)	19,415,251 (5.8%)
Free and reduced lunch	Not applicable	Not applicable	219 (31.5%)	6,349 (32.4%)	356,433/863,227 (41.3%)	Not available^3^
Social vulnerability index^5^						
High (0.67–1)	184 (18.8%)	150 (21.4%)	34 (12.2%)	6 (15.0%)	448 (27.1%)	27,912 (33.2%)
Medium (0.34–0.66)	297 (30.4%)	230 (32.9%)	67 (24.1%)	13 (32.5%)	587 (35.5%)	28,746 (34.2%)
Low (0–0.33)	497 (50.8%)	320 (45.7%)	177 (63.7%)	21 (52.5%)	617 (37.2%)	26,666 (31.7%)

1 Data obtained from Missouri Department of Elementary and Secondary Education for 2022.

2 2022: American Community Survey 1-Year Estimates.

3 Category not available in the data source.

4 American Indian NH, Native Hawaiian or Pacific Islander NH, and other NH accounted for < 1% of School KIDS participants.

5 Social Vulnerability Index (SVI) tertiles were calculated using the census-tract level U.S. SVI data, pulled 9 January 2024, from CDC/ATSDR SVI Data and Documentation Download | Place and Health | ATSDR. For NCKSD students, Missouri and the United States population columns, SVI tertiles were calculated using SVI scores from all census tracts within the respective geographical area as the denominator, representing the proportion of census tracts with high, medium, or low SVI scores within that area.

The 978 participants resided in 713 unique houses and 192 households had ≥2 School KIDS participants. In total, 29 (3.0%) participants withdrew from School KIDS with reasons including participant request or moving outside of the NKCSD during the study period.

### Symptom surveys associated with surveillance testing

Of the 5,538 symptom surveys sent to participants before scheduled surveillance testing, 4,069 (73.5%) surveys were completed with the majority (*n* = 2,726; 67%) documenting no symptoms in the preceding 7 days (Table 2). The most frequently reported symptoms were nasal congestion and runny nose. ARI symptoms were reported on 1,348 (33.1%) surveys, including 501 (12.3%) reporting symptoms as resolved and 846 (20.8%) reporting ongoing symptoms (one survey did not indicate whether symptoms had resolved). A corresponding surveillance specimen was obtained for 3,589 (88.2%) of completed surveillance surveys.

**Table 2:** Prevalence of respiratory symptoms in School KIDS participants by survey type.^1^

	Surveillance survey (*N* = 4,069)	On-demand survey (*N* = 722)	Convalescent survey (*N* = 707)
Nasal congestion	832 (20.4%)	544 (75.3%)	301 (42.6%)
Runny nose	690 (17.0%)	510 (70.6%)	276 (39.0%)
Cough	615 (15.1%)	462 (64.0%)	215 (30.4%)
Sore throat	405 (10.0%)	366 (50.7%)	128 (18.1%)
Fever	140 (3.4%)	153 (21.2%)	43 (6.1%)
Shortness of breath	56 (1.4%)	74 (10.2%)	32 (4.5%)
Wheezing	42 (1.0%)	48 (6.6%)	23 (3.3%)
Other symptoms^2^	–	1 (0.1%)	–
No recent symptoms^3^	2,726 (67.0%)	–	288 (40.7%)

1 Totals may be >100% as symptoms are >1 symptom may have been present.

2 Only asked on the on-demand symptom survey.

3 Only asked on the surveillance and convalescent surveys as on-demand testing required ≥1 respiratory symptom present at the time of testing.

### Surveillance testing

**Figure 3:**
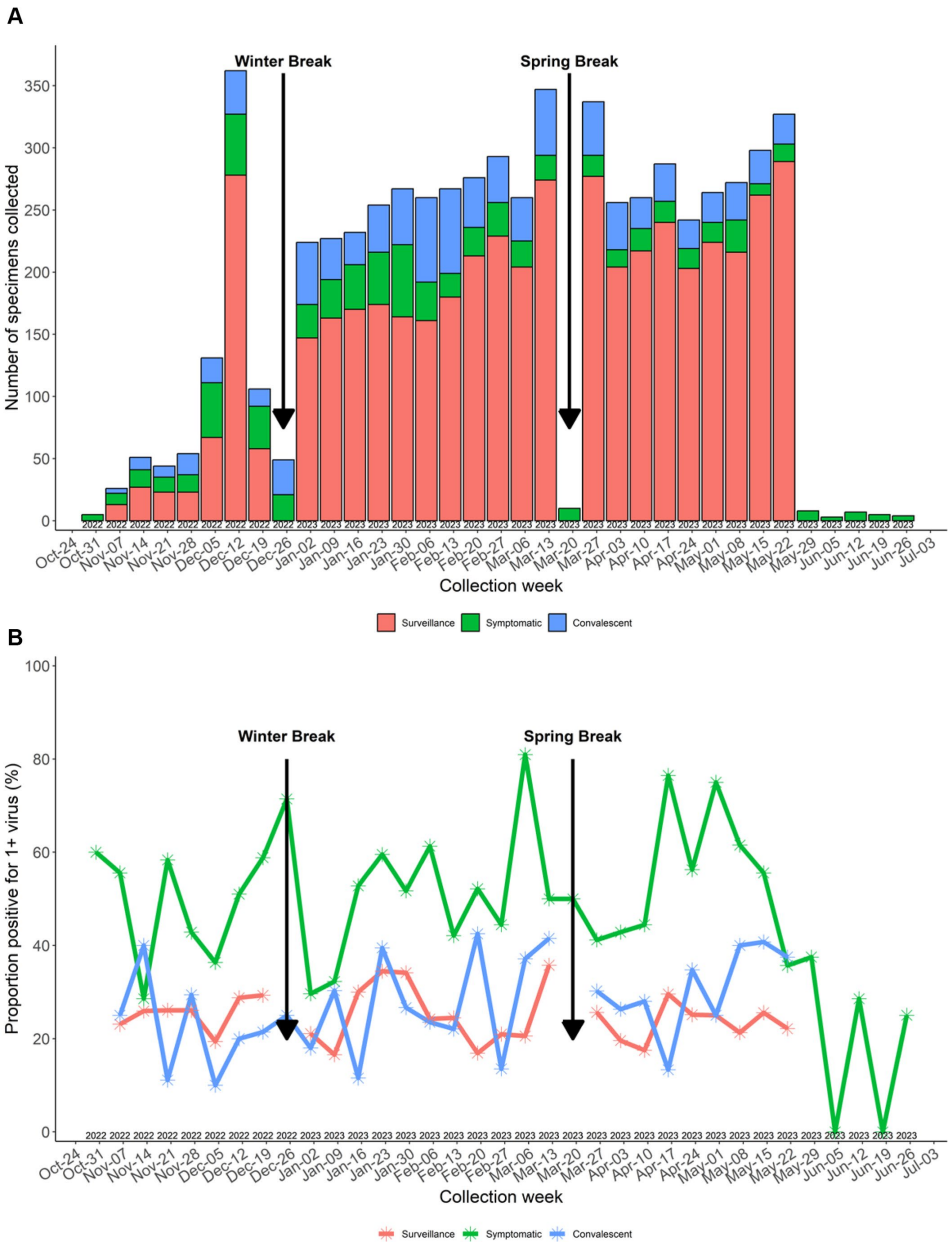
**(A)** Number of specimens collected by week. **(B)** Specimen virus positivity by week. Surveillance specimens not collected during winter break and spring break.

Of 5,252 surveillance tests attempted, 4,705 (89.6%) specimens were obtained. The number of surveillance tests scheduled (*n* = 5,252) did not match the number of symptom surveys sent (*n* = 5,538) due to changes in schedules after surveys were deployed but prior to the study team arriving at the school to perform surveillance testing (e.g., notification of a class field trip or participant submitted an interim on-demand symptomatic test). Of 547 specimens that were not collected, reasons included participant absent (*n* = 403, 73.7%), participant at school but not available (*n* = 103, 18.8%), unknown reason (*n* = 23, 4.2%), and participant refusal (*n* = 18, 3.2%). A total of 876 (98.3%) enrolled participants provided ≥1 surveillance specimen during the study with a median of six (IQR 4–7) surveillance specimens per participant. The number of surveillance specimens collected by week is displayed in Figure 3 showing that the number of specimens collected was relatively consistent each week when school was in session.

### Symptom surveys associated with on-demand symptomatic and convalescent testing

Symptom surveys were completed for all 722 (100%) on-demand symptomatic tests. The most frequently reported symptoms (Table 2) were nasal congestion (*n* = 544, 75.3%), runny nose (*n* = 510, 70.6%), and cough (*n* = 462, 64.0%). Of the 883 convalescent surveys sent to participants prior to scheduled convalescent testing, 707 (80.1%) were completed.

### On-demand symptomatic and convalescent testing

A total of 722 on-demand symptomatic swabs were submitted by 377 participants, with 235 students submitting 434 (60.1%) swabs and 142 staff submitting 288 swabs (39.9%) [median 1 (IQR 1–2) test/participant for both groups]. Of the 722 swabs submitted, 512 (70.9%) were obtained outside of the school setting. These were returned using either school drop-off (*n* = 130, 25.4%), CMKC Urgent Care drop-off (*n* = 192, 37.5%), or home courier (*n* = 190, 37.1%). The remaining 210 (29.1%) were obtained at school. Participants reported a median of 2 (IQR 1–3) days of symptoms at the time of test submission.

A total of 535 (74.1%) and 362 (50.0%) on-demand symptomatic specimens had at least one and two convalescent specimens, respectively, obtained in the subsequent 2 weeks for a total of 897 convalescent tests.

### Viruses detected

**Figure 4:**
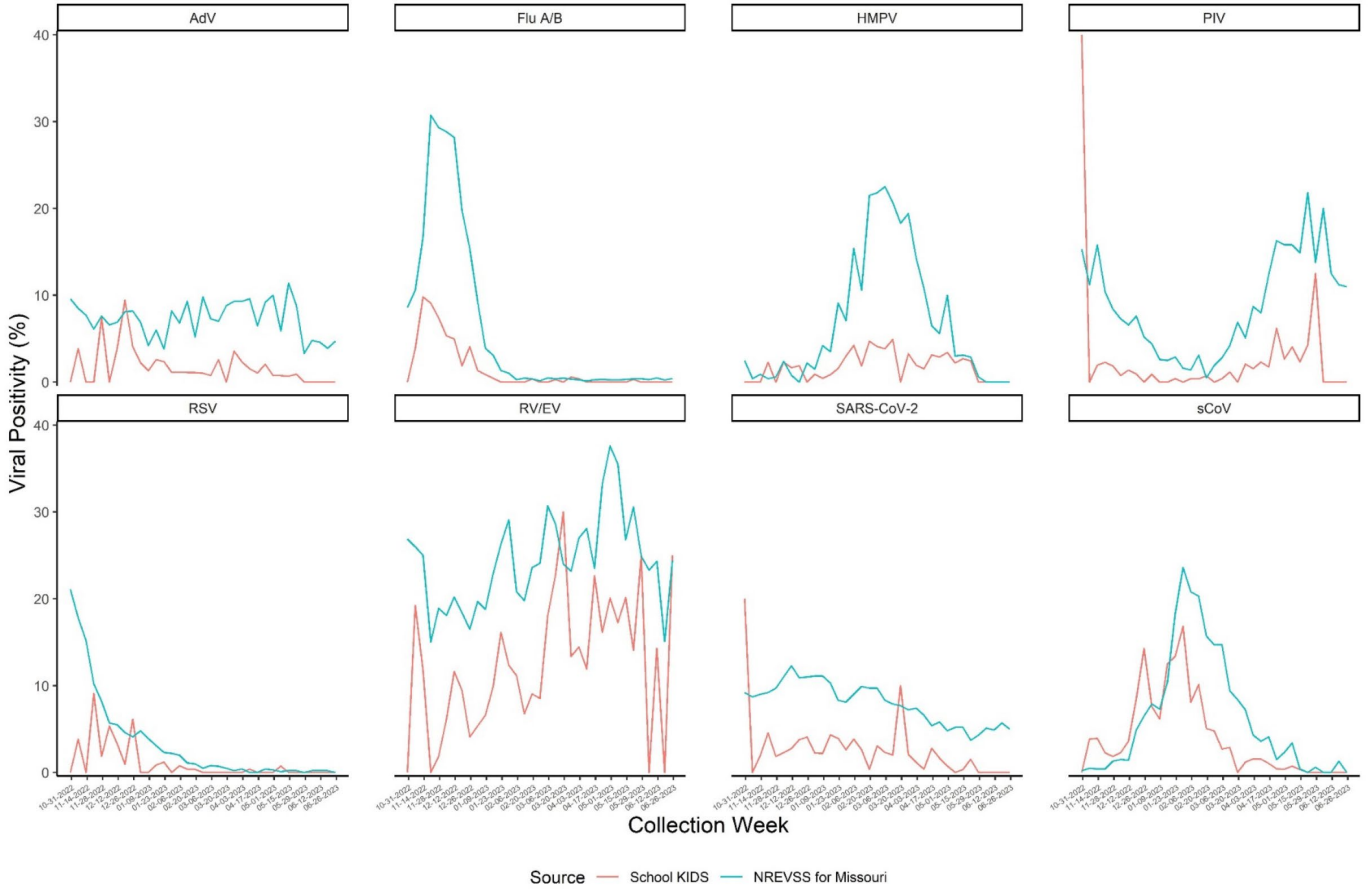
NRVESS state-level data was extracted on December 19, 2023, and results from July 2022 onward are preliminary. All results presented here are from nucleic acid amplification tests which represent >90% of the diagnostic tests reported to NREVSS. The last three weeks of data may be less complete. For more information on NREVSS, please visit http://www.cdc.gov/surveillance/nrevss. Only data during the study time frame of October 31, 2022-June 30, 2023 were used. SARS-COV-2: Severe acute respiratory syndromic coronavirus type 2 Flu: Influenza virus types (e.g., types A, B) are combined here but reported by type and subtype depending on the testing capabilities of each contributing laboratory. RSV: Respiratory Syncytial Virus. Types A and B are reported but are combined in this report. RV/EV: Rhinovirus or Enterovirus. These results are generally clinically indistinguishable and reported to NRVESS in a combined category. PIV: Parainfluenza virus types 1 through 4 are combined for this visual. However, laboratories report these data individually. sCoV: Human coronavirus types HKU1, OC43, 229E and NL63 are combined for this visual. However, laboratories report these data individually. AdV: Adenovirus, includes all adenovirus detections reported to NREVSS from respiratory specimen results (e.g., nasal pharyngeal swabs). There over 100 adenovirus types. Most commercial laboratory test do not distinguish type without further identification. HMPV: Human metapneumovirus types A and B are not reported separately from NREVSS.

Viral testing was performed on 6,315 out of 6,324 (99.9%) specimens collected; 9 specimens did not undergo viral testing due to insufficient samples. Of the tested specimens, 1,778 (28.2%) were positive for any respiratory virus, including 175 (2.8%) that were positive for ≥2 viruses. The viruses detected varied over time; trends in specific virus positivity over time in School KIDS mirrored state-wide trends in NREVSS (Figure 4). Overall, RV/EV was the most frequently detected virus in 969 (15.3%) specimens, followed by sCoVs, which were detected in 320 (5.1%) specimens. Specimens from pre-KG and elementary school students (44.4 and 34.6%, respectively) were more frequently positive than specimens from high school students and staff (20.9 and 20.5%, respectively), and the detection of specific viruses varied by age (Table 3). For example, adenovirus was detected in 8.9% of pre-KG swabs, but only 0.5 and 0.6% of high school students and staff, respectively. SARS-CoV-2 was detected in 3.9% of staff swabs, as compared to <2% of elementary, middle, and high school student swabs.

**Table 3:** Virus detections in all School KIDS specimens stratified by age group.^1^

	Total *n* = 6,315	Pre-kindergarten *n* = 180	Elementary school *n* = 2,671	Middle school *n* = 885	High school *n* = 641	Staff *n* = 1938
Any virus detected^2^	1778 (28.2%)	80 (44.4%)	924 (34.6%)	243 (27.5%)	134 (20.9%)	397 (20.5%)
AdV	135 (2.1)	16 (8.9%)	90 (3.4%)	14 (1.6%)	3 (0.5%)	12 (0.6%)
Flu A	58 (0.9%)	1 (0.6%)	32 (1.2%)	8 (0.1%)	6 (0.9%)	11 (0.6%)
Flu B	4 (<0.1%)	0 (0.0%)	0 (0.0%)	3 (<0.1%)	0 (0.0%)	1 (<0.1%)
HMPV	174 (2.8%)	12 (6.7%)	87 (3.3%)	22 (2.5%)	15 (2.3%)	38 (2.0%)
PIV 1–4	107 (1.7%)	8 (4.4%)	56 (2.1%)	16 (1.8%)	10 (1.6%)	17 (0.9%)
RSV	46 (0.7%)	2 (1.1%)	24 (0.9%)	7 (0.8%)	8 (1.2%)	5 (0.3%)
RV/EV	969 (15.3%)	36 (20.0%)	547 (20.5%)	146 (16.5%)	67 (10.5%)	173 (8.9%)
SARS-CoV-2	148 (2.3%)	4 (2.2%)	43 (1.6%)	14 (1.6%)	11 (1.7%)	76 (3.9%)
sCoV	320 (5.1%)	11 (6.1%)	159 (6.0%)	38 (4.3%)	25 (3.9%)	87 (4.5%)
≥2 viruses detected	175 (2.8%)	9 (5.0%)	110 (4.1%)	24 (2.7%)	9 (1.4%)	23 (1.2%)
No virus detected	4,537 (71.8%)	100 (55.6%)	1747 (65.4%)	642 (72.5%)	507 (79.1%)	1,541 (79.5%)

1 Viruses tested include adenovirus (AdV), human metapneumovirus (HMPV), influenza A and B (Flu), parainfluenza viruses 1–4 (PIV), respiratory syncytial virus (RSV), rhinovirus/enterovirus (RV/EV), SARS-CoV-2, and seasonal coronaviruses 229E, HKU1, NL63, and OC43 (sCoV).

2 Specific viruses are not mutually exclusive and may total more than the denominator due to multiple detections in some specimens.

**Figure 5:**
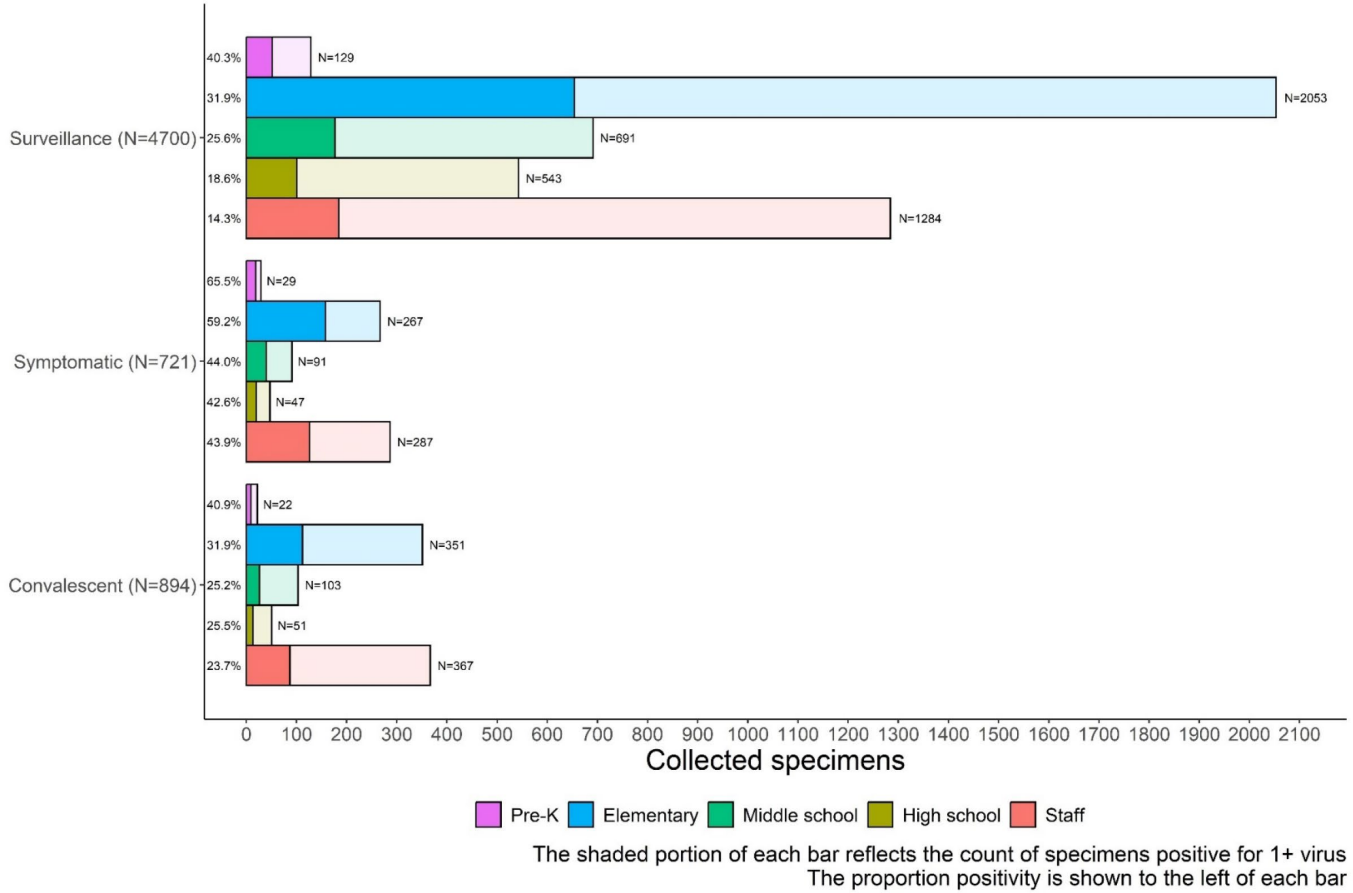
School KIDS specimen type and percentage positive for one or more viruses by age group. Adv, Viruses tested include adenovirus; HMPV, human metapneumovirus; Flu, influenza A and B; RSV, syncytial virus; RV-EV, rhinovirus/enterovirus; sCov, SARS-CoV-2, and seasonal coronaviruses 229E, HKU1, NL63, and OC43.

Of the 4,705 surveillance tests, 1,168 (24.9%) were positive for ≥1 virus, with the highest percent positivity in pre-kindergarten students (52 out of 129 specimens, 40.3%) (Figure 5). Surveillance specimen positivity peaked at 35.8% during 13–19 March 2023 (Figure 3B). On-demand symptomatic swabs were submitted throughout the school year, including school breaks and summer school (Figure 3). On-demand specimen percent positivity peaked at 81.0% during the week of 6–12 March 2023 (Figure 3B). A total of 363 (50.3%) specimens had ≥1 virus detected with the highest percent positivity in pre-kindergarten (19 out of 29, 65.5%) and elementary school (158 out of 267, 59.2%) students (Figure 5).

### Vaccine verification

All participants had attempted vaccine verification through the state registries, and the majority (713 out of 978, 72.9%) of participants consented to allow the team to contact providers to obtain vaccination records. Overall, 179 (18.3%) participants received the 2022–23 COVID-19 bivalent vaccination, and 399 (40.8%) received the 2022–23 seasonal influenza vaccine. Vaccine receipt varied by age group (Table 4).

**Table 4:** COVID-19 (any and bivalent) and seasonal influenza (2022–2023) vaccination status among School KIDS participants.

	Total (*n* = 978)	Pre-kindergarten (*n* = 43)	Elementary school (*n* = 404)	Middle school (*n* = 143)	High school (*n* = 110)	Staff (*n* = 278)
Any COVID-19 vaccine^1^	630 (64.4%)	18 (41.9%)	215 (53.2%)	95 (66.4%)	74 (67.3%)	228 (82.0%)
Bivalent COVID-19 vaccine	179 (18.3%)	5 (11.6%)	44 (10.9%)	24 (16.8%)	27 (24.5%)	79 (28.4%)
Influenza vaccine (2022–2023)	399 (40.8%)	24 (55.8%)	166 (41.1%)	66 (46.2%)	42 (38.2%)	101 (36.3%)

1 Any COVID-19 vaccine type by any manufacturer.

## Discussion

Community-based surveillance for ARI and respiratory viruses is needed to better understand the full epidemiology of circulating viruses. Schools represent a unique congregate community setting, where children spend most of their day and are exposed to multiple other people. However, a paucity of data exists related to baseline viral detection in the school setting, despite schools being associated with respiratory viral outbreaks. In this study, we highlight methodologic successes for performing community-based respiratory viral surveillance in the school setting. By partnering with the school district for recruitment, we were able to enroll a diverse population, representative of the local and regional population. With over 98% of participants providing at least one specimen and a median of 6 specimens over 6 months of enrollment, longitudinal assessment of viral detection in participants is feasible. Furthermore, we had an overall return rate of 74.4% of all surveys, allowing for the assessment of symptoms at the time of specimen collection.

Although overall viral detection ranged from 21 to 44% depending on age group, on average almost one-quarter of surveillance specimens, which were obtained at school, had a detectable respiratory virus. Schools are congregate settings, and therefore, it is recommended that respiratory viral prevention strategies are implemented routinely which can include respiratory etiquette, hand hygiene, and staying up to date on vaccinations. As viral circulation rates increase, additional strategies may be considered beyond general infection prevention (e.g., the use of well-fitted masks and improved ventilation). Understanding baseline viral circulation in schools is important to prioritize layered infection prevention strategies during periods of increased viral surges in the community. SchoolKIDS virus-specific circulation data mirrored state-wide laboratory-based data (from NRVESS), suggesting that schools could supplement existing passive surveillance platforms by contributing symptom data in non-medically seeking individuals and serve as an effective place to perform active community-based surveillance. More surveillance data collection and temporal analyses are needed to understand the timing of increased school respiratory virus transmission data relative to community transmission data and/or clinical surveillance data, but it could potentially provide earlier detection for novel pathogens and signal respiratory virus surges.

Although schools are an important community setting in which to understand baseline respiratory viral circulation, they present unique challenges when integrating viral surveillance, including protection of minors, absence of parents/legal guardians at the time of specimen collection, and the need to minimize educational disruptions. Before the COVID-19 pandemic, school-based ARI surveillance and specimen collection were not well-described, and during the COVID-19 pandemic, multiple barriers were identified related to student and staff participation and testing for SARS-CoV-2 surveillance programs (12). Here, we present the successful implementation of a school-based surveillance program that could serve as a roadmap to understanding the burden of non-medically attended ARI and baseline viral circulation.

Accurately capturing the presence or absence of symptoms at the time of respiratory testing is important to categorize ARI. In this program, we used text and email surveys to communicate with parents and participants before testing. These notifications served two purposes: (1) notifying caregivers that their child would be tested and (2) assessing recent symptoms associated with testing. A recent meta-analysis demonstrated an average online broad survey response rate of 44% (22). By keeping the survey short and using mixed modalities for delivery (23), we were able to obtain a good response rate to capture symptom status.

School-based surveillance has been infrequently performed; however, we incorporated available data in the design of School KIDS. Previous surveillance studies have demonstrated the success of non-invasive specimen collection techniques (e.g., buccal swabs) (24). In addition, weekly school-based surveillance may be less efficient, leading us to implement monthly surveillance. This study was designed to accommodate schools and families while minimizing disruption in education, which led to successful implementation, specimen collection, and survey responses. A key element to the success of the study was the close partnership between the study staff and the school district. The school district provided feedback on the study design and coordinated with the study team ways to maximize participation and minimize school day disruption. School district stakeholders served as the trusted messengers to present the study to students and staff. Participation was emphasized as optional. To identify days of the week and times of the day when surveillance could be performed with minimal disruptions, School KIDS coordinated with the school district and school nurses. Close collaboration with the school district allowed for the successful collection of approximately 90% of scheduled surveillance tests. For on-demand symptomatic testing, offering a variety of specimen collection locations (i.e., home or school) and delivery methods (i.e., courier and two drop-off locations) provided multiple opportunities to collect specimens during ARI episodes. In addition to flexibility, a key component of the success was the partnership among study staff, participants, and the school district. Participants, parents, and school staff had access to the study team by email and phone during regular school hours when questions arose related to testing or test results. Not only were participants compensated for their time but also the district was provided a stipend for their time and effort in this project. Finally, data were provided back to participants. Participants were notified if they had COVID-19, influenza, or another virus. COVID-19 and influenza were specifically relayed as they were considered actionable (e.g., available treatments and isolation measures) by the study team. Multiple curious participants contacted the study staff to determine what non-COVID-19, non-influenza virus was detected from their on-demand symptomatic specimen, and these data were provided to individuals with links to CDC webpages with plain language information about symptoms, prevention, and treatment of the viruses. Participant-level data were not provided to the school district, and isolation was at the discretion of the participant. Aggregate, de-identified data were provided to the district.

This study does have some limitations. Overall enrollment was <5% of the district student population; however, participants were representative of the school district, and some schools had greater than 10% of overall student enrollment. Study onset and specimen collection started in the middle of the school year and did not capture a full year of respiratory illness and viral circulation. Peak RSV was likely missed due to the timing of surveillance start and peak RSV circulation (25). Surveillance days were missed because of inclement weather and school holidays although testing was rescheduled when feasible, and surveillance testing was performed on 95 out of 121 (78.5%) days when school was in session during the study period. School KIDS required several study personnel to conduct testing in the schools. Having a large study team readily available to perform testing can be cost prohibitive for some school-based studies; however, study staff minimized the burden of testing on school nurses and staff members. Specimens were collected using anterior self-administered nasal swabs, and the sample could have been sub-optimal based on the collection technique, although surveillance specimen collection was witnessed by a member of the study staff or school nurse, and samples were tested by highly sensitive molecular assays that also detect housekeeping genes to ensure adequate specimen collection. Finally, not all completed surveillance symptom surveys were associated with specimen collection (e.g., absent participant or rescheduled test collection due to interim on-demand symptomatic testing), and not all surveillance specimen collections were associated with a completed survey as this was not a requirement for surveillance testing.

## Conclusion

School-based surveillance for ARI and respiratory viruses is feasible, and specimen collection can occur in the school setting. In future, schools may serve as a unique environment to study community-based ARI, viral transmission in congregate settings, and the effectiveness of layered strategies to prevent respiratory virus transmission.

## Data availability statement

The raw data supporting the conclusions of this article will be made available by the authors, without undue reservation.

## Ethics statement

The studies involving humans were approved by the Children’s Mercy Kansas City and Centers for Disease Control and Prevention. The studies were conducted in accordance with the local legislation and institutional requirements. The ethics committee/institutional review board waived the requirement of written informed consent for participation from the participants or the participants’ legal guardians/next of kin because Both IRBs provided a non-research determination due to public health surveillance.

## Author contributions

JS: Conceptualization, Data curation, Funding Acquisition, Supervision, Writing – original draft, Writing – review & editing. TC: Writing – original draft, Writing – review & editing. CY: Project administration, Writing – original draft, Writing – review & editing. BL: Formal analysis, Visualization, Writing – review & editing. DB: Writing – review & editing. AS: Writing – review & editing. OA: Writing – review & editing. HK: Writing – review & editing. JP: Writing – review & editing. AD: Writing – review & editing. ST: Writing – review & editing. RS: Writing – review & editing. JG: Conceptualization, Data curation, Funding acquisition, Supervision, Writing – original draft, Writing – review & editing.
