# Supplementary figures and images for: School knowledge of infectious diseases in schools: conducting surveillance and on-demand, symptomatic respiratory viral testing in a large pre-kindergarten–12th grade school district
Source: Front Public Health. 2024 Jul 23;12:1408281. doi: 10.3389/fpubh.2024.1408281 (PMC11300224; doi:10.3389/fpubh.2024.1408281)

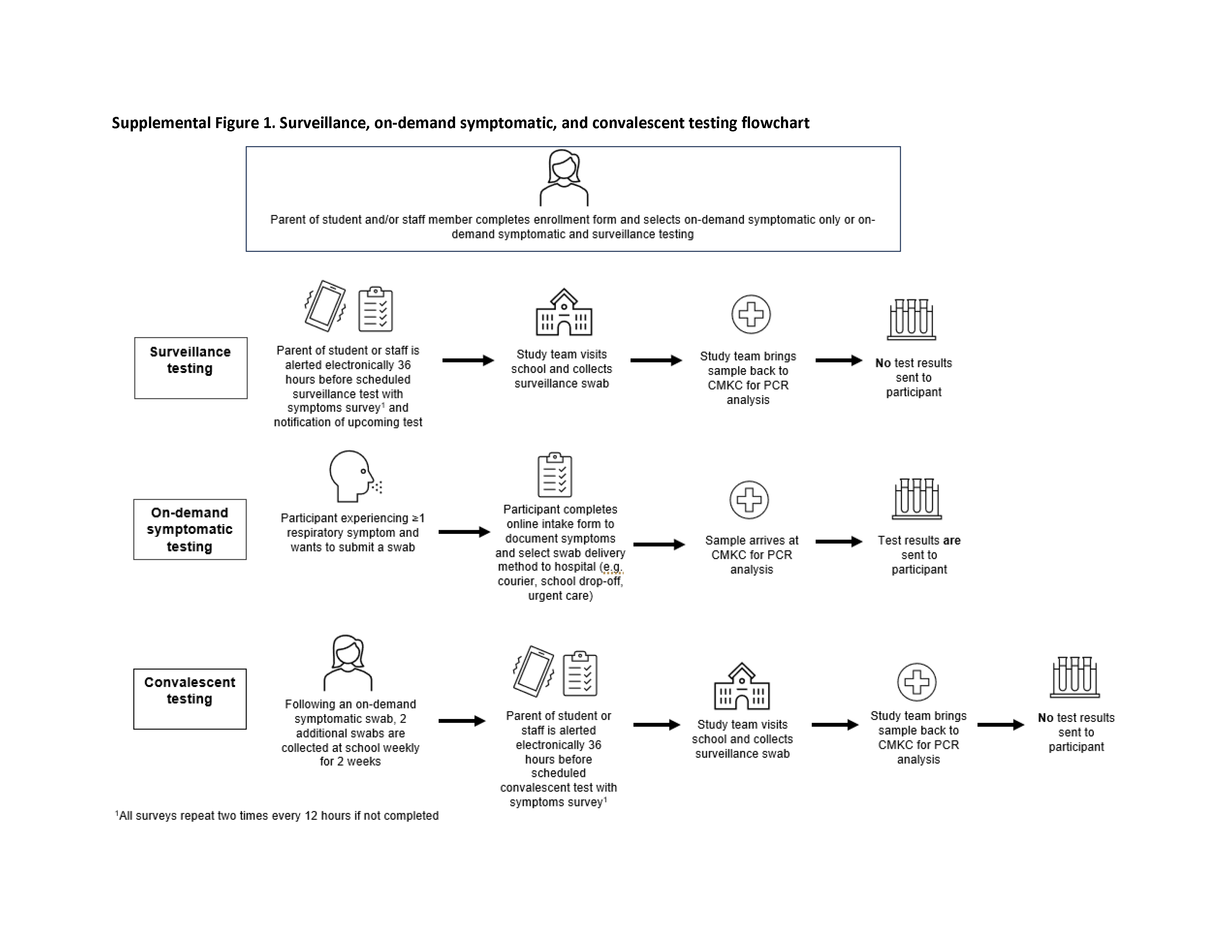

Supplement: Supplementary file 1 [file Image_1.TIFF]

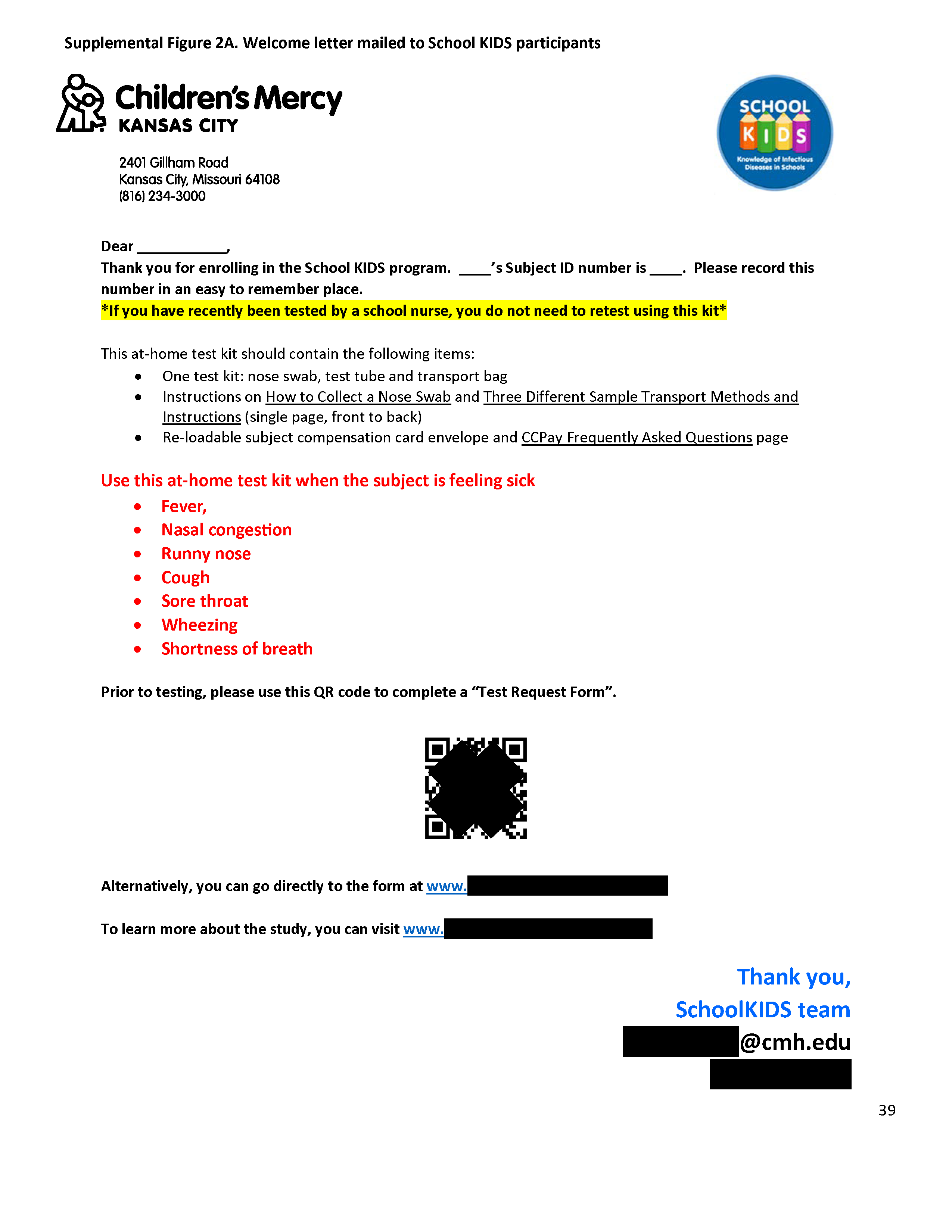

Supplement: Supplementary file 2 [file Image_2.TIFF]

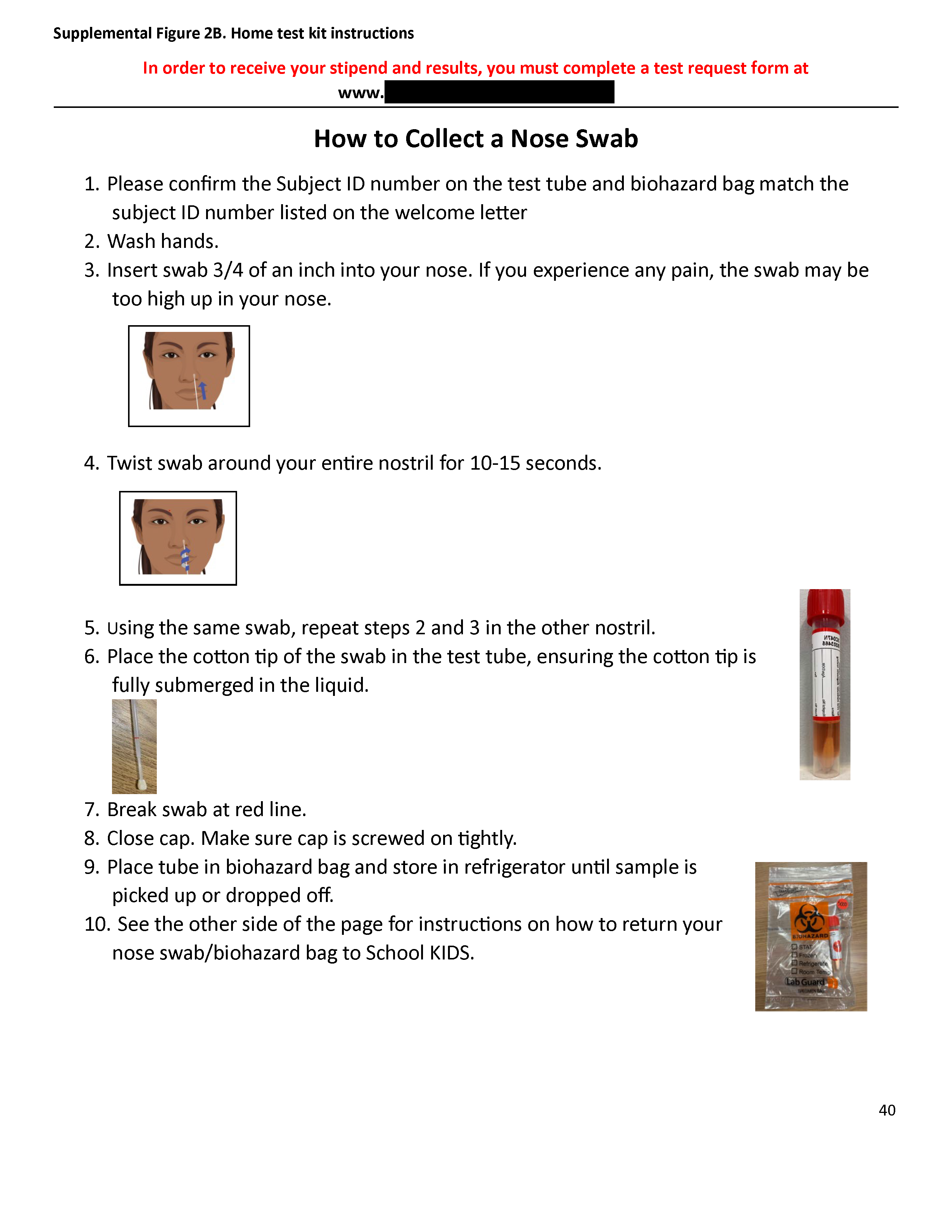

Supplement: Supplementary file 3 [file Image_3.TIF]

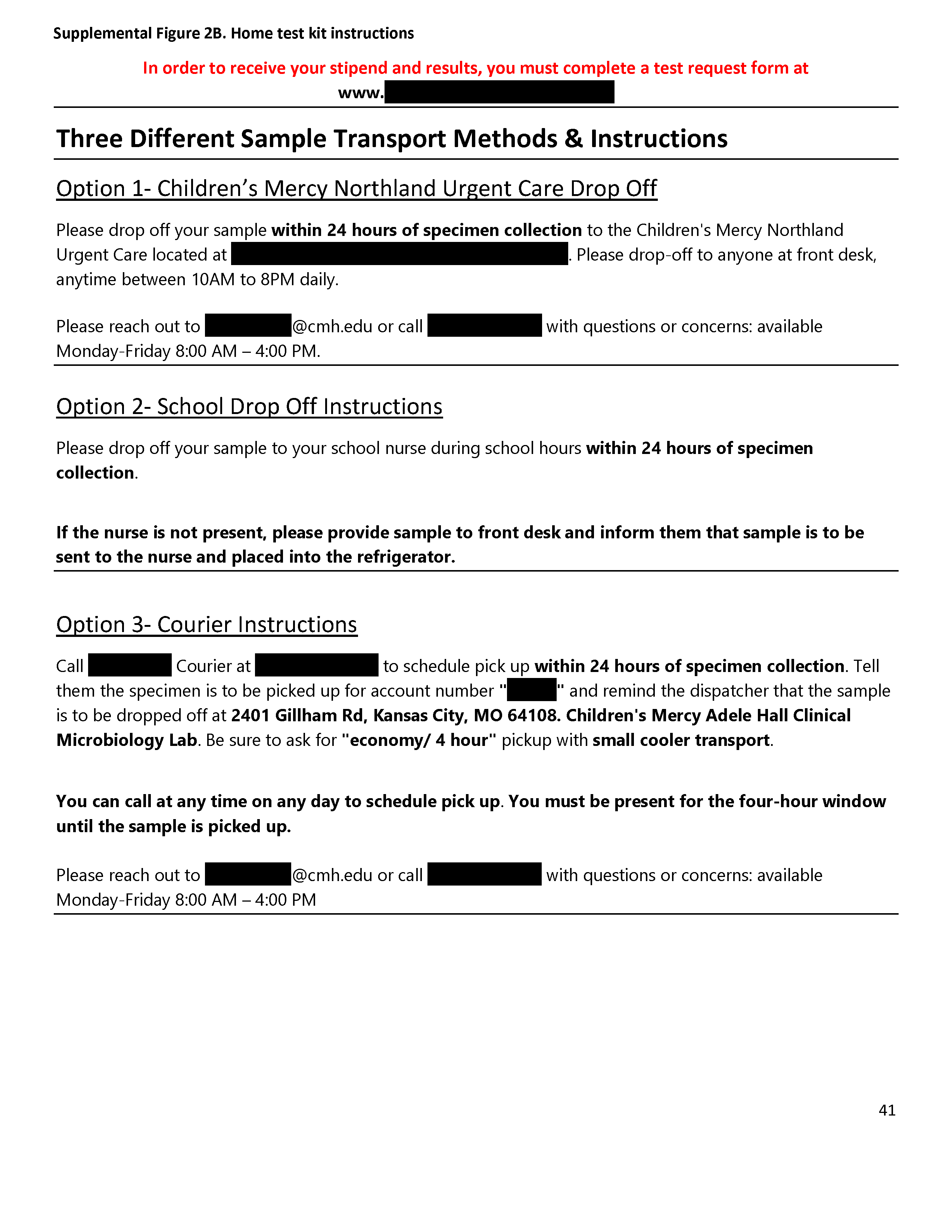

Supplement: Supplementary file 4 [file Image_4.TIF]
